# Behavioral factors associated with multiple-type HPV genital infections: data from a cross-sectional study in young women in Brazil

**DOI:** 10.1186/s12978-021-01244-2

**Published:** 2021-10-10

**Authors:** Natália Luiza Kops, Juliana Caierão, Marina Bessel, Jaqueline Driemeyer Correia Horvath, Carla Magda Domingues, Adele Schwartz Benzaken, Luisa Lina Villa, Flávia Moreno Alves de Souza, Gerson Fernando Mendes Pereira, Eliana Marcia Wendland

**Affiliations:** 1 Hospital Moinhos de Vento, Porto Alegre, Brazil; 2 Department of Analysis, Faculty of Pharmacy, Federal University of Rio Grande do Sul, Porto Alegre, Brazil; 3 National Immunization Program, Ministry of Health, Brasilia, Brazil; 4 Doctor Heitor Vieira Dourado Tropical Medicine Foundation, Molecular Biology Laboratory Manaus, Manaus, Brazil; 5 Faculdade de Medicina, and Instituto do Câncer do Estado de São Paulo (ICESP), Universidade de São Paulo, São Paulo, Brazil; 6 Department of Chronic Conditions and Sexually Transmitted Infections, Ministry of Health, Brasília, Brazil; 7 Graduate Program in Health Sciences and Pediatrics, Federal University of Health Science of Porto Alegre, Rua Ramiro Barcelos 910, Porto Alegre, CEP 90035-004 Brazil

**Keywords:** HPV, Epidemiology, Sexual health, Infectious diseases, Young adults

## Abstract

**Objectives:**

To investigate the pattern of multiple *human papillomavirus* (HPV) infections and associated factors in young women who access the Brazilian public health care system to better understand the characteristics of multiple HPV infections, a critical issue in this era of multivalent vaccines.

**Methods:**

This was a cross-sectional, multicenter study with sexually active unvaccinated women (16–25 years old) from 119 primary Brazilian healthcare centers between September 2016 and November 2017. Cervical samples were collected by trained health professionals, and HPV detection was performed in a central laboratory by Linear Array.

**Results:**

Of the 5268 women, 33.00% (95% CI 31.07–34.92) had multiple infections. At least one type of high-risk HPV was present in 85.50% of all multiple infections. All HPV types were detected more frequently in association with other types than alone. Young individuals who were single or in a casual relationship and those who had more than one sexual partner in the past year were more likely to have multiple infections.

**Conclusions:**

In this work, a high rate of multiple HPV infections among unvaccinated young adults tended to increase due to certain risk factors. Such data can provide insight for decision makers in the development of public policies regarding HPV prevention.

## Plain Language Summary

Understanding the characteristics of multiple infections is critical in the era of HPV multivalent vaccines for the prevention of cervical carcinomas. Therefore, in this cross-sectional study, we aimed to investigate the pattern of multiple HPV infections and associated factors in 5,268 sexually active unvaccinated women (16–25 years old) who access the Brazilian public health care system. Cervical samples were collected by trained health professionals, and HPV detection was performed in a central laboratory by Linear Array. A total of 33.00% (95% CI 31.07–34.92) had multiple infections (60.43% of the HPV-positive sample). The number of HPV types in a multiple infection ranged from 2 to 14 different types. The viral types more frequently identified were HPV 16 and 52. All HPV types were detected more frequently in association with other types than alone. The incidence of multiple infections was 1.29 times higher in single than in married or cohabitating participants. Women who had two or more partners in the last year also had higher rates of multiple infections than those who had fewer than two sexual partners. In conclusion, a high prevalence of multiple infections prior to the national HPV immunization program was observed, especially with the increase in less safe behavior factors.

## Resumo

Compreender as características de infecções múltiplas é fundamental na era das vacinas multivalentes contra o HPV para a prevenção do cancer de colo de útero. Portanto, neste estudo transversal, tivemos como objetivo investigar o padrão de infecções múltiplas de HPV e fatores associados em 5.268 mulheres sexualmente ativas não vacinadas (16-25 anos) que acessam o sistema público de saúde brasileiro. Amostras cervicais foram coletadas por profissionais de saúde treinados. A detecção do HPV foi realizada em um laboratório central por Linear Array. Ao todo, 33,00% (95% IC 31,07–34,92) das participantes tiveram infecções múltiplas (60,43% entre aquelas com HPV positivo). O número de tipos de HPV em uma infecção múltipla variou de 2 a 14. Os tipos virais mais frequentemente identificados foram 16 e 52. Todos os tipos de HPV foram detectados mais frequentemente em associação com outros tipos do que isoladamente. A incidência de infecções múltiplas foi 1,29 vezes maior em participantes solteiros do que em casados. Mulheres que tiveram duas ou mais parcerias sexuais no último ano também tiveram taxas mais altas de infecções múltiplas do que aquelas que tiveram menos de duas parcerias. Em conclusão, foi observada uma alta prevalência de infecções múltiplas antes do Programa Nacional de Imunização contra o vírus, especialmente naqueles que apresentam comportamentos menos seguros.

## Introduction

Cervical cancer kills approximately 300,000 women yearly, particularly middle-aged women and those living in lower-resource settings [1]. In Brazil, this is the fourth most frequent cancer among women despite being largely preventable through vaccination [2]. The percentage of multiple human papillomavirus (HPV) infections found in invasive cervical carcinomas has greatly increased, although this is a monoclonal disease that should be caused by only one HPV type [3]. HPV types 16 and 18 account for ~ 70% of all cervical cancer cases worldwide, while other types account for an additional 20% [4].

The first HPV studies seldom detected multiple infections. This is likely due to the characteristics of early diagnostic tests. The shift toward highly sensitive assays has, however, affected HPV findings in the most recent epidemiological studies in a way that cannot be ignored [5]. Notably, coinfections with multiple HPV types are very common in sexually active men and women [6]. In addition to the viral genotype, many other factors could lead to cervical carcinoma, such as viral persistence, age and immune status [7]. The contribution of HPV genotypes individually or pooled based on the severity of cervical neoplasia is unclear [1, 8, 9] but seems to be associated with persistent HPV infections [9, 10].

Indeed, because some studies have demonstrated higher rates of abnormal cervical cytology in women with multiple-type infections [6, 11, 12], it is important to understand the biological and epidemiological characteristics of these specific HPV infections. Some studies suggest that multiple types of infections occur more frequently than would be expected by chance alone [6]. This indicates that acquisition of different HPV types is, thus, not independent and that shared factors may be the explanation for the increased frequencies of coinfections [13]. At the same time, other studies, controlling for the lifetime number of sexual partners, age, and type-specific HPV prevalence, have demonstrated that the number of cases with multiple-type infections is small [14, 15]. Accordingly, multiple genital HPV infections are notably associated with aspects of sexual behavior, such as the number of sexual partners [16, 17]. These findings reinforce that cervical cancer and less safe behaviors are often linked, reflecting an opportunity, especially for the young adult population, which has a high prevalence of sexually transmitted infections (STIs).

Understanding the characteristics of multiple infections is critical in the era of HPV multivalent vaccines. To contribute to this issue, this study was developed with the objective of investigating the pattern of multiple HPV infections and associated factors in young women who access the Brazilian public health care system.

## Methods

### Study population

This study analyzed the data collected in the POP-Brazil study (*Prevalence of Human Papillomavirus in Brazil*). This is the first nationwide study aimed at determining the prevalence of HPV infections (and their types) among sexually active women aged 16–25 years [18]. Participants were enrolled from 119 primary health care units of all 27 Brazilian capitals between September 2016 and November 2017. Individuals were recruited through their reference health units, which were selected using criteria related to the representativeness of the health districts of each capital and the ability to collect and store samples. The exclusion criteria were as follows: pregnant women; those who had undergone hysterectomy or trachelectomy; and participants who had cervical intraepithelial neoplasia grade 2 or higher. Participants who did not complete the questionnaire, participants who answered the questionnaire but did not provide genital samples, and participants who had already received the HPV vaccine were excluded from the analysis. As part of the POP-Brazil study, all participants were interviewed based on a structured questionnaire. Data regarding social and demographic aspects (current age < 22 or ≥ 22 years old, race/color, relationship status), sexual behavior (the number of partners in the past and in the last 5 years, age at first sexual intercourse, condom use in the last intercourse and type of sex practiced), drugs, alcohol, and tobacco use were collected. To characterize sexually transmitted infections, we asked participants if they had ever been diagnosed with HIV, syphilis, gonorrhea, and/or herpes. Additionally, participants were invited to undergo an HIV rapid test. Individuals who reported having a sexually transmitted infection (STI) or who had two positive rapid test results at the time of the interview were considered positive for STI. Details on the instruments used to collect these variables are available elsewhere [19].

Cervical samples were collected by trained health professionals using a Qiagen HC2 DNA collection device according to the manufacturer’s instructions. The swabs were placed in Digene Specimen Transport Medium (Qiagen), stored at controlled room temperature (15–25 °C) and shipped to a central laboratory weekly where the samples were aliquoted and stored at − 80 °C until processing.

Samples were concentrated by centrifugation, and DNA was extracted by using a robotic system (MagNA Pure LC 2.0; Roche) in a central laboratory, which utilizes magnetic beads to purify DNA. Roche Linear Array® was used to perform HPV detection and typing, as previously described [18]. The test detects 37 types of HPV simultaneously [20]. The assay used incorporated β-globin as an internal control for sample amplification. Whenever necessary, the presence of HPV-52 was confirmed using a specific real-time PCR assay.

The study was approved by the Ethics Committee on Human Research (Moinhos de Vento Hospital – protocol no. 1607032). All participants provided written consent.

### Statistical analysis

HPV results were grouped as high-risk HPV types (HR-HPV) (16, 18, 31, 33, 35, 39, 45, 51, 52, 56, 58, 59, 68) and others (6, 11, 26, 40, 42, 53, 54, 61, 62, 64, 66, 67, 69, 70, 71, 72, 73, 81, 82, 82v, 83, 84, 89) according to the manufacturer’s instructions. Young adults infected with more than one HPV type were considered positive with multiple infections.

Descriptive analysis was used to characterize the study population. Categorical variables were summarized using absolute frequencies and percentages, while continuous variables were analyzed using means and confidence intervals (CIs). Chi-square tests and analysis of variance (ANOVA) were performed to detect differences between groups. The answer option “I don’t know” was considered a missing value. The percentages of all variables were calculated after applying a weighting variable to the sample, which was constructed according to the distributions of the populations of Brazilian capitals by sex in the age group of interest in 2010 according to the Demographic Census of the Brazilian Institute of Geography and Statistics (IBGE).

Initially, Poisson regression with robust variance analysis was conducted to examine factors associated with multiple HPV infections compared with single infection, adjusting for confounders. These analyses provided a prevalence ratio (PR) as a measure of association. For the multivariate analyses, a theoretical framework was structured with the variables associated with multiple infections, discriminating hierarchical blocks [11, 21–24]: personal characteristics (Model 1), relationship and smoking status (Model 2), and sexual behavior (Model 3). The hierarchical model is an available alternative in epidemiological studies with a large number of covariates [25].

In the next step, from the full model, the variables significantly associated with HPV were defined as less safe behavior factors for the infection. Finally, the prevalence of negative, single, and multiple HPV infections was analyzed according to the presence of these selected factors (none, one, two or three factors) throughout a multiple comparison test for proportions in a cross tabulation. The Cochran-Armitage trend test was also used to determine how the prevalence of multiple HPV infections changes according to the presence of these factors. Statistical analysis was performed using SAS software (version 9.4, Statistical Analysis System, SAS Institute Inc., Cary, N.C.), and statistical significance was defined as p < 0.05.

The sample size (7935) was based on the main objective of the POP-Brazil Study, which was to establish the prevalence of HPV in Brazil. It was considered an HPV prevalence of 30%—estimated by a systematic review that analyzed infection in the cervix [26]. It was deliberately equal in all regions to maximize diversity in less populated areas.

## Results

The mean age of the 5,268 women included in the study was 21.80 years (CI 21.69–21.90). The distribution of individuals according to single and multiple HPV infections is shown in Table 1.

**Table 1:** Characteristics of young adult women between HPV groups

	Single HPV n (%)	Multiple HPV n (%)	P value
Age (%)			< 0.001
16–21 years	588 (34.72)	1064 (65.28)	
22–25 years	534 (46.24)	665 (53.76)	
Race/color (%)			0.692
White	246 (41.77)	407 (58.23)	
Black	180 (35.89)	279 (64.11)	
Brown	659 (39.60)	993 (60.40)	
Other (Asiatic, indigenous)	33 (44.91)	44 (55.09)	
Relationship status (%)			< 0.001
Single or without partner	224 (31.65)	406 (68.35)	
In casual relationships	401 (35.37)	808 (64.63)	
Married or living together	485 (50.12)	494 (49.88)	
Divorced or widowed	12 (40.33)	21 (59.67)	
No. of sexual partners in the last year (%)			< 0.001
< 2	841 (43.07)	1089 (56.93)	
≥ 2	254 (31.51)	579 (68.49)	
No. of sexual partners in the last 5 years (%)			< 0.001
≤ 1	308 (50.18)	294 (49.82)	
2 or 3	400 (40.58)	622 (59.42)	
≥ 4	301 (32.29)	611 (67.71)	
Tobacco use			0.712
Nonsmoker	775 (40.17)	1203 (59.83)	
Smoker	139 (36.79)	200 (63.21)	
Ex-smoker	208 (39.08)	326 (60.92)	
Drug use (%)			0.764
Yes	271 (38.84)	443 (61.16)	
No	851 (39.80)	1286 (60.20)	
Alcohol consumption (%)			< 0.001
Yes	791 (36.17)	1266 (63.83)	
No	331 (47.73)	463 (52.27)	
Condom use in the last intercourse (%)			0.226
Yes	398 (37.30)	654 (62.70)	
No	720 (40.80)	1067 (59.20)	
Sexually transmitted infection^c^			0.670
Yes	139 (38.64)	202 (61.36)	
No	925 (40.43)	1410 (59.57)	
Type of sex (%)			0.287
Exclusively vaginal	304 (43.23)	446 (56.77)	
Other sex types excluding vaginal	19 (47.64)	19 (52.36)	
Vaginal and other sex types	779 (38.58)	1204 (61.42)	

POP-Brazil Study

Data are shown as absolute frequencies and percentages

**Fig. 1:**
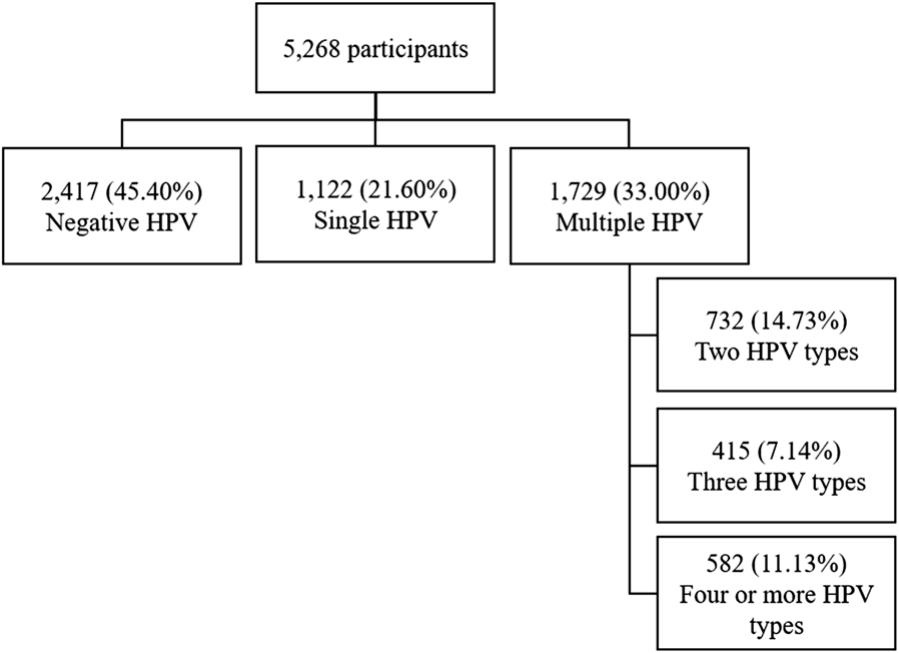
Types of HPV infection in young adult women in the POP-Brazil Study (2016–2017). The percentages were calculated after applying a weighting variable to the sample

A total of 1,729 women [33.00% (95% CI 31.07–34.92)] had multiple infections (60.43% of the HPV-positive sample; 1729/2851), ranging in number of types from 2 to 14. The majority had two HPV types, but 11.13% had four or more HPV types (Fig. 1). Of participants with multiple infections, 84.50% (1456/1729) had at least one high-risk (HR-HPV) type (data not shown).

**Fig. 2:**
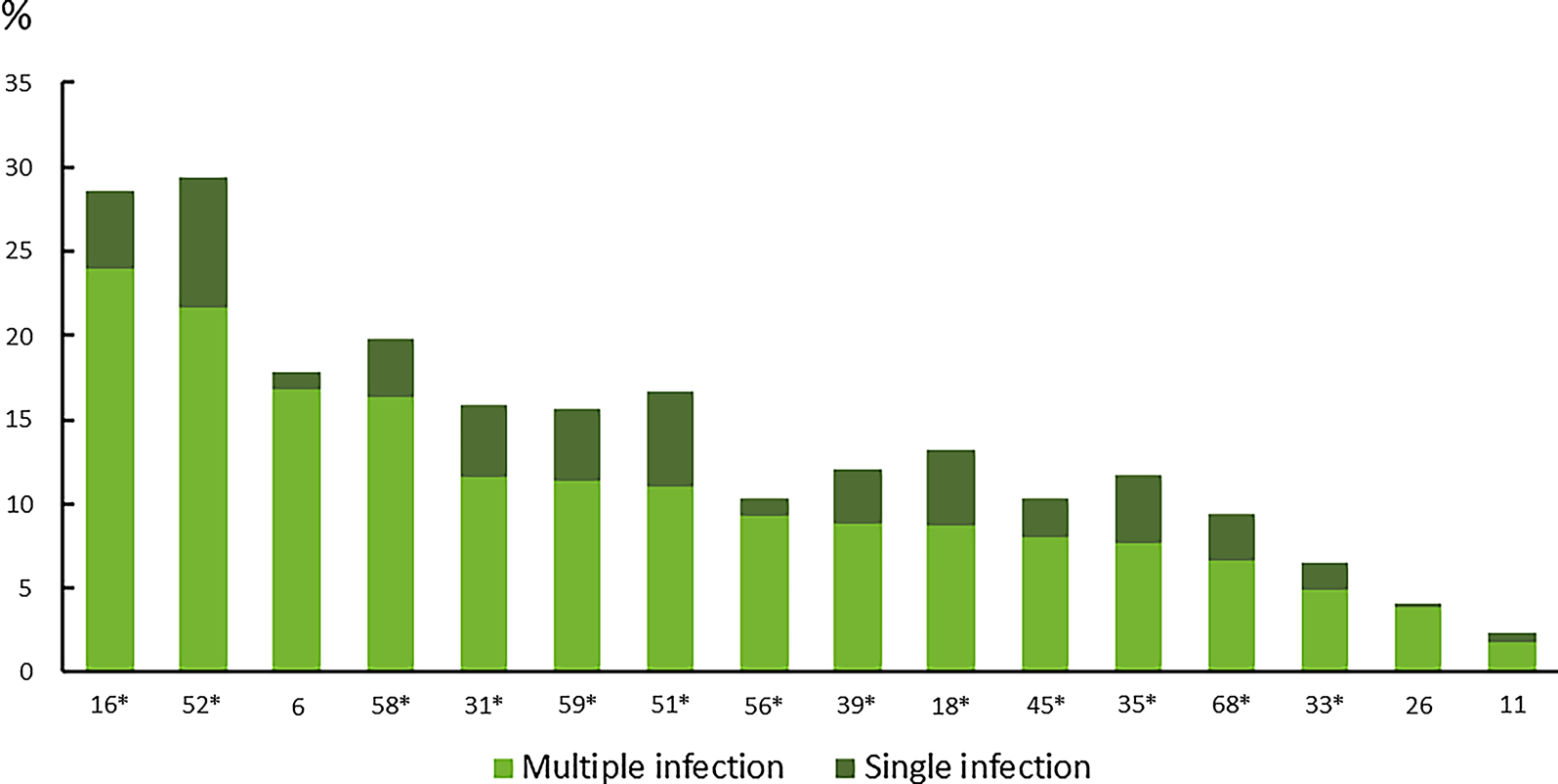
Prevalence of the most common HPV genotypes by single and multiple infections. *High-risk HPV type

The viral types more frequently identified in participants with multiple infections were HPV 16 (411, 23.97%), HPV 52 (364, 21.67%), HPV 6 (266, 16.81%), and HPV 58 (262, 16.30%). Individually, all HPV types were detected more frequently in association with other types than alone (Fig. 2).

### Characteristics by different groups of infection

The prevalence of multiple infections changes significantly with age (p < 0.001). The prevalence of multiple infections was 65.28% and 53.76% among women younger and older than 22 years, respectively (Table 1). Single participants, those with a higher number of partners (last year and last 5 years), smokers, and those who consumed alcohol had higher rates of multiple HPV infection. The mean age at first sexual intercourse of participants with multiple infections was 15.48 (15.39–15.56) years, without significant differences between the infection groups (data not shown). There was no difference in the HPV multiple infection rate according to self-declared race/skin color (p = 0.692).

When we compared participants with multiple infections with those with single infections, being 22 years or older was a protective factor in all multivariate models (Table 2). The incidence of multiple infections was 1.29 times higher among participants who were single [PR 1.29 (CI 95% 1.12–1.48)] and 1.20 times higher among those involved in casual relationships [PR 1.20 (CI 95% 1.06–1.36)] compared with the incidence among married participants or those or who were living together. Women who had two or more partners in the last year had higher rates of multiple infections (PR 1.13; 95% CI 1.02–1.25) than those who had fewer than two sexual partners.

**Table 2:** Multivariate analyses of factors associated with multiple HPV infections compared with single infections

	Model 1	Model 2	Model 3
	Prevalence ratio (95% confidence interval)		
Age			
16–21 years	1	1	1
22–25 years	0.82 (0.75–0.91)	0.85 (0.77–0.93)	0.84 (0.75–0.93)
Race/color			
White	1	1	1
Black	1.10 (0.95–1.27)	1.09 (0.94–1.25)	1.11 (0.95–1.29)
Brown	1.03 (0.92–1.17)	1.05 (0.93–1.18)	1.05 (0.93–1.20)
Other	0.94 (0.67–1.33)	0.93 (0.65–1.32)	0.89 (0.60–1.34)
Relationship status			
Single or without partner		1.34 (1.19–1.52)	1.29 (1.12–1.48)
Dating or casual		1.26 (1.13–1.42)	1.20 (1.06–1.36)
Married or living together		1	1
Divorced/widowed		1.17 (0.78–1.76)	1.10 (0.73–1.67)
Smoking status			
Nonsmoker		1	1
Smoker		1.05 (0.91–1.20)	1.04 (0.89–1.21)
Ex-smoker		1.01 (0.90–1.13)	1.02 (0.91–1.15)
No. of sexual partners in the last year			
< 2			1
≥ 2			1.13 (1.02–1.25)
Sexually transmitted infection			1.00 (0.87–1.14)
Age at first sexual intercourse			
< 14 years			1.02 (0.89–1.17)
≥ 14 years			1
Type of sex			
Exclusively vaginal			1
Other sex types excluding vaginal			0.86 (0.46–1.62)
Vaginal and other sex types			1.06 (0.95–1.19)
Condom use in the last intercourse			1.00 (0.90–1.10)

Poisson regression with robust variance

Model 1: age and race/color

Model 2: Model 1 + relationship status and smoking status

Model 3: Model 2 + number of sexual partners in the last year, presence of sexually transmitted infection, age at first sexual intercourse, type of sex practices and condom use in the last intercourse

### Presence of less safe behavioral factors

**Fig. 3:**
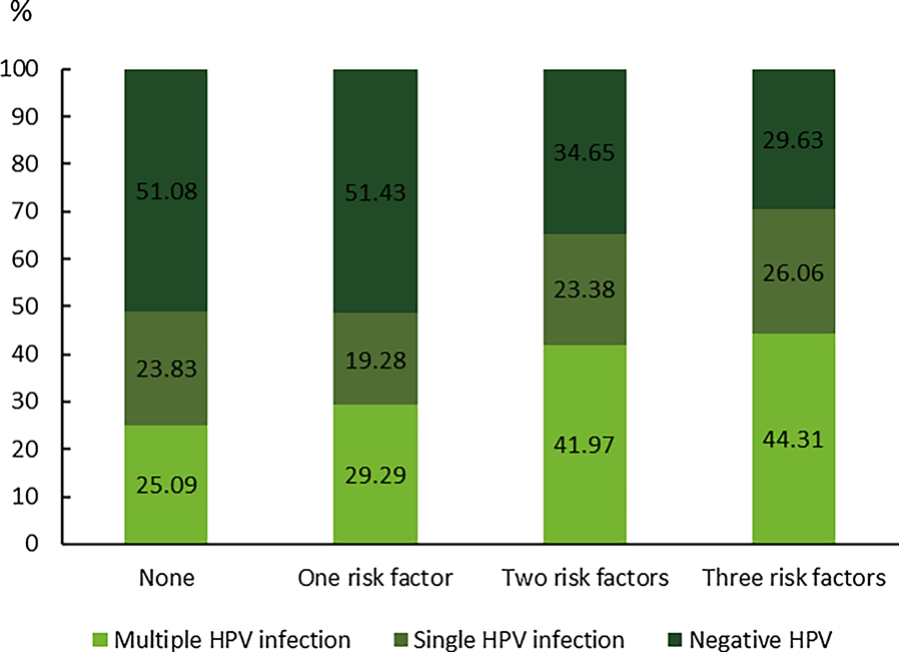
Prevalence of single and multiple infections according to the presence of less safe behaviors. The less safe behaviors analyzed were younger age (< 22 years), single or in casual relationships, and two or more partners in the last year

We analyzed the prevalence of infections according to the presence of the less safe behavioral factors identified in Table 2 (younger age, single or dating relationship, and two or more partners in last year). In a trend analysis, the prevalence of multiple infections increased significantly as the number of less safe behaviors increased (p < 0.001). The prevalence of multiple infections was significantly higher than that of single HPV infection for all women with one (29.29% vs. 19.28%), two (41.97% vs. 23.38%) and three behaviors (44.31% vs. 26.06%), in addition to those with no behavioral factors (Fig. 3).

## Discussion

This large nationwide sample of sexually active young adult Brazilian women showed that the occurrence of multiple HPV infections was high, especially HR-HPV, and more common than that of single infections. The two most commonly detected HR-HPV types in multiple infections were HPV 16 and 52. Young women, single or dating status, and a higher number of sexual partners were the factors most associated with multiple HPV infections. The prevalence of multiple infections had an upward trend with the number of less safe behavior factors.

More than 30% of all participants had multiple HPV infections. Other studies with larger age ranges showed a prevalence varying between 19.0 and 43.2%. In the United States, the prevalence was 19.0% (participants aged 18–65 years) [6], compared with 28.5% in Colombia (aged 12–19 years)[21], 31.3% in Greece (aged 18–71 years) [27], 33.5% in Canada (aged 13–86 years) [28], 41.9% in Sweden (aged 12–45 years) [15], and 43.2% in Costa Rica (aged 18–25 years) [11]. However, in another Brazilian study (2,075 women; mean age of 33 years), the prevalence of multiple infections was much lower even among participants with cytologic abnormalities (3% among those with normal cytology, 7% among woman with high-grade squamous intraepithelial lesion, 10% among those with atypical squamous cells of undetermined significance, and 23% among those with low-grade squamous intraepithelial lesion), but it was also age-dependent, which could explain the differences compared with our findings [13]. It is possible that infection in the early years of sexual life leads not only to a higher prevalence but also to a higher frequency of HPV types. Viral clearance of HPV 16 is lower at younger ages [29] and is associated with viral load, which could be higher in sexually naïve women.

HPV 16 was the most frequent genotype found in multiple infections, which is in accordance with previous studies involving sexually active young adults [3, 5, 9, 21, 27, 30–32]. Similar to HPV 16, all other types were more likely to be detected as part of a multiple infection rather than as single infections [31], which is different from the results reported by Argyri and Dunne [27, 33]. This may be due to the age of this study’s participants (16–25 years), as participants under the age of 22 years were associated with multiple infections. In the United States, for example, the prevalence of single HPV infections is more common, but the study population comprised only females aged 14 to 59 years [33]. The same result was observed in a Greek study (women aged 18 to 71) [27]. There seems to be a tendency of HPV types to cluster significantly with the genetic similarity of L1 regions [14]. In the present study, both of the more prevalent HR-HPV types were detected in patients with multiple infections (16 and 52) belonging to the Alpha 9 species.

Our results are consistent with the literature, which demonstrates inverse associations of a higher prevalence of multiple HPV with younger age [22]. High evidence was also seen for women who were single or dating compared with married women or women who live with someone. Sexual contact with different types of partners is already a well-established risk factor for the prevalence of HR-HPV [34] and appears to be a risk factor for multiple infections as well [11, 21, 24, 34]. Other factors, such as race/skin color and condom use, were not associated with multiple infections. The more risk factors the participant had, the higher the prevalence of multiple infections. No other studies were found that evaluated the trend between risk factors and multiple infections.

Despite knowing that the prevalence of multiple HPV infections is high, it remains unclear whether this is a higher risk factor for the persistence of HPV and for cervical lesions than single infections [7, 8]. Wentzensen [32] and Trottier [12] showed that multiple HR infections were not associated with an increase in squamous intraepithelial lesions, and Chaturvedi [11] and the SUCCEED study [35] found a significantly increased risk of cervical intraepithelial neoplasia in coinfections with multiple genotypes. Recently, Oyervides-Muñoz also found an association between multiple HPV infections and high viral loads and infection persistence, a requirement for developing cervical cancer [9]. Some studies have shown that women coinfected with both HR- and low-risk HPV have a reduced risk for future invasive squamous cervical cancer compared with that for women infected with HR-HPV alone [36]. Apparently, some specific combinations could synergistically or antagonistically interact and affect the risk of cancer [37], but further clinical studies must be designed to determine the mechanism of these combinations.

Regardless of whether HPV types in multiple infections are randomly chosen, understanding the characteristics of these types of infections and the groups of risk factors is critical in the era of HPV vaccination. In Brazil, the quadrivalent vaccine (types 6, 11, 16 and 18) has been used in the Public Health System as a primary measure for cervical cancer prevention since 2014 [38]. Initially, girls aged 11 to 13 years were targeted, followed by those aged 9 to 13 years in 2015 and 9 to 14 years in 2017; at that time, boys aged 11 to 14 years and other populations at risk, such as HIV-positive people, oncologic patients and people with solid organ or bone marrow transplantation, were also considered. Post-vaccination data may corroborate the discussion on quadrivalent vaccine effectiveness in the context of multiple-type HPV rates. This is the first large study that is demographically and geographically diverse and includes all Brazilian capitals that use a sensitive testing method that allows the identification of multiple HPV types simultaneously. Additionally, we were able to provide important data regarding the behavior of HPV multiple infections according to the number of less safe behavioral factors.

Some limitations of this study should be noted. Although we recruited participants in different settings, this was a cross-sectional study with a convenience sample. However, the sociodemographic and behavioral characteristics are similar to those of the young Brazilian population, allowing inferences for the entire population in this age range. Additionally, we do not have information on HPV immunogenicity and were not able to assess concurrent versus acquired infections, considering that immune responses following concurrent acquisition of multiple HPV infections could be different from natural acquired infections.

In conclusion, we observed a high prevalence of multiple infections, especially HR-HPV, prior to the national HPV immunization program. This prevalence tended to increase with a higher number of less safe behavioral factors. Such data can provide insight for decision makers in the development of public policies regarding HPV prevention.

## Data Availability

The original data from the survey are available upon request.

## Abbreviations

ANOVA: Analysis of variance
CI: Confidence interval
HPV: Human papillomavirus
HR: High risk
POP-Brazil: Prevalence of Human Papillomavirus in Brazil
PR: Prevalence ratio
SAS: Statistical Analysis System
STI: Sexually transmitted infection
